# Enhancing Type 2 Diabetes Management: Exploring the Synergistic Impact of Vestibular Exercise and Yoga

**DOI:** 10.7759/cureus.48539

**Published:** 2023-11-08

**Authors:** Athira MS, Sheela Joice P P, Mohan Varughese, Suresh Babu Sayana

**Affiliations:** 1 Department of Research, Vinayaka Missions Research Foundation (Deemed University), Salem, IND; 2 Department of Physiology, Vinayaka Missions Kirupananda Variyar Medical College, Salem, IND; 3 Department of General Medicine, Believers Church Medical College Hospital, Thiruvalla, IND; 4 Department of Pharmacology, Government Medical College and General Hospital, Suryapet, IND

**Keywords:** anthropometric parameters, metabolic parameters, yoga, vestibular stimulation, type 2 diabetes

## Abstract

Background

Type 2 diabetes management often necessitates a multifaceted approach encompassing metabolic and anthropometric parameters. This study explores the potential of vestibular stimulation activities and yoga as complementary strategies in improving the health of individuals with type 2 diabetes.

Methods

A total of 180 participants were divided into three groups: vestibular exercises alone, yoga alone, and a combined group undertaking both interventions. Various metabolic parameters including fasting blood sugar (FBS), postprandial blood sugar (PPBS), HbA1c (glycosylated hemoglobin), cholesterol, lipid profile, and blood pressure, alongside anthropometric parameters like body mass index (BMI), body fat percentage, waist, hip circumference, waist-hip ratio, and arm and thigh circumference, were measured at baseline and after three months of intervention.

Results

Vestibular exercises and yoga, when practiced separately, demonstrated significant reductions in FBS (p < 0.01 for both). Both interventions were also effective in improving PPBS control (p < 0.01). Yoga led to a greater decrease in HbA1c compared to the control group (p < 0.01), suggesting a stronger impact on long-term glucose regulation. Vestibular exercises reduced total cholesterol and low-density lipoprotein (LDL) significantly (p < 0.01), while yoga additionally lowered triglycerides and increased high-density lipoprotein (HDL) cholesterol (p < 0.01), and notably reduced systolic blood pressure (p < 0.01). In terms of anthropometric parameters, the yoga group exhibited a significant reduction in BMI (p < 0.01), with the combined group showcasing the most substantial reduction (p < 0.01). Both yoga and the combined group achieved significant reductions in body fat percentage (p < 0.01), waist and hip circumferences (p < 0.01), and arm and thigh circumferences (p < 0.01). The combined intervention showed a borderline significant decrease in waist-hip ratio (p = 0.074).

Conclusion

Vestibular stimulation activities and yoga, whether practiced separately or together, have a beneficial impact on various metabolic and anthropometric parameters in individuals with type 2 diabetes. Combining these interventions appears to yield the most pronounced improvements, offering a holistic approach to enhancing type 2 diabetes management. These findings emphasize the potential of incorporating vestibular stimulation activities and yoga into diabetes care programs to promote overall health and well-being in individuals with type 2 diabetes.

## Introduction

Type 2 diabetes mellitus (T2DM) stands as one of the most prevalent and challenging chronic health conditions globally [1]. Characterized by insulin resistance and impaired glucose regulation, T2DM poses significant health risks, including cardiovascular complications, neuropathy, and kidney disease. Its increasing prevalence, often associated with sedentary lifestyles and unhealthy dietary habits, has necessitated the exploration of holistic and non-pharmacological approaches to its management [1,2]. One such approach, gaining recognition for its potential benefits, combines vestibular stimulation activities and yoga practices to address both metabolic and anthropometric aspects of diabetes management [3,4].

The vestibular system, located within the inner ear, plays a crucial role in maintaining balance and spatial orientation [5,6]. Vestibular stimulation activities involve specific movements and exercises that activate this system [7]. While traditionally associated with balance and coordination, recent research has unveiled its potential to influence metabolic parameters. Additionally, yoga, an ancient mind-body practice, is known for its diverse health benefits, including stress reduction, improved flexibility, and enhanced cardiovascular health [8,9]. When integrated into diabetes management, yoga has demonstrated positive effects on glycemic control, lipid profiles, and blood pressure [10].

The notion of combining vestibular stimulation activities with yoga is founded on the belief that the synergy of these two interventions may yield more substantial health improvements than either approach alone [11]. Vestibular stimulation activities engage the body in controlled movements that challenge balance and spatial awareness, potentially enhancing insulin sensitivity and glucose utilization [12-15]. Yoga, on the other hand, offers a comprehensive approach to overall well-being, addressing not only metabolic parameters but also body composition, mental health, and stress management [13,14]. The combination of these interventions aims to create a holistic framework for T2DM management.

The present study seeks to investigate the impact of vestibular stimulation activities and yoga practices, both individually and in combination, on various metabolic and anthropometric parameters in individuals diagnosed with T2DM. These parameters include fasting blood sugar (FBS), postprandial blood sugar (PPBS), HbA1c levels (glycosylated hemoglobin), lipid profiles, blood pressure, body mass index (BMI), body fat percentage, waist and hip circumferences, waist-hip ratio, and arm and thigh circumferences.

The aim of this study was to explore the synergistic effects of vestibular stimulation activities and yoga on T2DM management by assessing their impact on metabolic and anthropometric parameters. Specifically, the study aims to evaluate the individual effects of vestibular stimulation activities and yoga on metabolic parameters, including FBS, PPBS, HbA1c levels, and lipid profiles, as well as assess their individual effects on blood pressure (systolic and diastolic). Furthermore, the study seeks to analyze the individual effects of these interventions on various anthropometric parameters, including BMI, body fat percentage, waist and hip circumferences, waist-hip ratio, and arm and thigh circumferences, all within the context of individuals with type 2 diabetes. Ultimately, the study aims to investigate the combined impact of vestibular stimulation activities and yoga on these metabolic and anthropometric parameters, providing a comprehensive assessment of their potential benefits for diabetes management.

## Materials and methods

Selection of subjects

The subjects for this study were selected based on specific criteria aligned with the diagnosis of T2DM as per the World Health Organization (WHO) guidelines. All potential participants were presented with a comprehensive explanation of the study's objectives and scope prior to their inclusion. The study involved a total of 180 participants distributed among three intervention groups.

Study setting

The research was conducted at Believers Church Medical College Hospital in Kuttappuzha, Thiruvalla, Kerala. This hospital served as the primary location for subject recruitment, data collection, and the implementation of both vestibular stimulation activities and yoga interventions.

Inclusion criteria

The inclusion criteria included participants who voluntarily gave informed consent to participate in the study, subjects who were diagnosed with T2DM in accordance with WHO criteria, and the study included subjects from a range of gender and age groups.

Exclusion criteria

The exclusion criteria included participants with Type 1 diabetes mellitus, those unable or unwilling to participate in vestibular stimulation exercises, and those with severe medical conditions or comorbidities that could significantly affect the study's outcomes were all excluded from the study.

Interventions

Participants were categorized into three groups: vestibular exercises alone, yoga alone, and a combined group that underwent both interventions. It was a non-randomized trial. Vestibular stimulation activities encompassed a series of exercises targeting the vestibular system, including movements such as head rotation, leaning, and walking with specific head turns. Yoga sessions comprised practices including Anulom-vilom, Sun Salutation, Paschimothanasana, Poorvothanasasana, Ardha matsyendra asana, Sasankasan, Naukasana, Trikonasana, and Savasana. These interventions were implemented over a three-month period.

The parameters that were covered by the data collection were as follows: metabolic parameters include lipid profiles, HbA1c levels, FBS, and postprandial blood sugar (PPBS); systolic and diastolic blood pressure measurements were made; anthropometric measurements included measurement of arm and thigh circumferences, waist and hip circumferences, waist-hip ratio, body fat percentage, and body mass index (BMI).

Data analysis

Statistical analysis of the collected data utilized appropriate statistical tests such as t-tests and ANOVA to evaluate the influence of vestibular stimulation activities and yoga interventions on the metabolic and anthropometric parameters. The significance level was set at p < 0.05.

Ethical considerations

Ethical clearance for the study was obtained from the Institutional Ethical Committee (IEC) under study number IEC/2021/06/217. Informed consent was diligently obtained from all participants, ensuring strict adherence to ethical principles throughout the research process.

This study meticulously observed ethical principles, and the participation of all individuals was contingent upon obtaining informed consent. The research was executed in compliance with the guidelines and the approval of the Institutional Ethical Committee (IEC).

## Results

Vestibular exercises, when practiced alone, resulted in a significant reduction in FBS levels after three months (from an average of 157.75 to 151.12). This reduction was statistically significant with a p-value of less than 0.01 (Table 1).

**Table 1:** Baseline metabolic parameters after three months among the control group and vestibular exercise groups. FBS: fasting blood sugar, PPBS: post-prandial blood sugar, HBA1C: glycosylated hemoglobin, LDL: low-density lipoprotein, HDL: high-density lipoprotein, SBP: systolic blood pressure, DBP: diastolic blood pressure. Results are considered statistically significant if the p-value is less than 0.05 and highly statistically significant if the p-value is less than 0.01.

Parameters	Control (before) (n=60) mean±SD	Control (after) (n=60) mean±SD	T value	P value	Vestibular exercise (before) (n=60) mean±SD	Vestibular exercise (after) (n=60) mean±SD	T value	P value
FBS (mg/dL)	156.97± 17.69	155.95 ± 17.85	2.55	<0.05	157.75 ± 19.33	151.12 ± 17.69	8.81	<0.01
PPBS (mg/dL)	276.32 ± 36.10	277.22 ± 35.93	2.31	<0.05	276.52 ± 36.12	257.67 ± 36.19	14.22	<0.01
HBA1C (%)	9.14 ± 0.33	9.12± 0.34	2.08	<0.05	9.13 ± 0.339	9.05 ± 0.337	5.07	<0.01
Cholesterol (mg/dL)	230.27 ± 23.05	228.95 ± 23.74	1.09	>0.05	250.57 ± 23.44	216.58 ± 22.40	13.21	<0.01
Triglyceride (mg/dL)	166.85 ± 20.76	165.93 ± 20.89	3.44	<0.01	167.03 ± 20.96	158.53 ± 20.17	15.63	<0.01
LDL (mg/dL)	166.85 ± 22.85	115.93 ± 22.77	2.42	<0.05	117.33 ± 22.85	114.45 ± 22.40	7.68	<0.01
HDL (mg/dL)	36.47 ± 5.23	35.93 ± 5.66	2.65	<0.05	36.63 ± 5.66	35.90 ± 5.22	2.89	<0.01
SBP (mm Hg)	133.27 ± 9.22	132.18 ± 9.39	1.97	<0.05	133.53 ± 9.53	130.43 ± 9.02	7.94	<0.01
DBP (mm Hg)	86.57 ± 7.13	85.73 ± 6.01	3.05	<0.01	86.60± 7.15	85.73 ± 6.71	6.71	<0.01

Both the yoga group and the combined vestibular exercise and yoga group saw significant reductions in body fat percentage (p < 0.01). This suggests that both interventions contribute to reducing body fat (Table 2).

**Table 2:** Baseline anthropometric parameters after three months among control group and vestibular exercise. BMI: body mass index, SD: standard deviation. The results are considered statistically significant if the p-value is less than 0.05 and highly statistically significant if the p-value is less than 0.01.

Parameters	Control (before) (n=60) mean±SD	Control (after) (n=60) mean±SD	T value	P value	Vestibular exercise (before) (n=60) mean±SD	Vestibular exercise (after) (n=60) mean±SD	T value	P value
BMI (kg/m²)	27.40 ± 2.73	27.32 ± 2.54	1.21	>0.05	27.43 ± 2.57	27.13 ± 2.62	8.08	<0.01
Body fat %	28.11 ± 3.49	27.95 ± 3.47	0.479	>0.05	29.32 ± 4.41	27.95 ± 3.47	2.57	<0.01
Waist circumference (cm)	100.14 ± 6.47	99.99 ± 6.51	1.48	>0.05	100 ± 6.56	99.70 ± 6.29	2.65	<0.01
Hip circumference (cm)	103.63 ± 5.20	103.52 ± 5.15	1.49	>0.05	103.66 ± 5.19	102.86 ± 5.23	6.86	<0.01
Waist–hip ratio	0.966 ± 0.035	0.965 ± 0.035	0.37	>0.05	0.967 ± 0.0359	0.969 ± 0.0346	-0.952	>0.05
Arm circumference (cm)	13.94 ± 3.40	13.79 ± 3.45	1.19	>0.05	13.87± 3.417	13.32 ± 3.285	11.90	<0.01
Thigh circumference (cm)	22.22 ± 4.12	22.02 ± 4.16	2.11	<0.05	22.08 ± 4.183	21.01 ± 4.189	5.72	<0.01

Similarly, yoga alone also produced a noteworthy reduction in FBS (from an average of 157.03 to 138.87), with a highly significant p-value of less than 0.01. These findings indicate that both vestibular exercises and yoga have a beneficial impact on FBS levels in individuals with type 2 diabetes (Table 3).

**Table 3:** Baseline metabolic parameters after three months among the control group and yoga group. FBS: fasting blood sugar, PPBS: post-prandial blood sugar, HBA1C: glycosylated hemoglobin, LDL: low-density lipoprotein, HDL: high-density lipoprotein, SBP: systolic blood pressure, DBP: diastolic blood pressure, SD: standard deviation. The results are considered statistically significant if the p-value is less than 0.05 and highly statistically significant if the p-value is less than 0.01.

Parameters	Control (before) (n=60) mean±SD	Control (after) (n=60) mean±SD	T value	P value	Yoga (before) (n=60) mean±SD	Yoga (after) (n=60) mean±SD	T value	P value
FBS (mg/dL)	156.97± 17.69	155.95 ± 17.85	2.55	<0.05	157.03 ± 17.61	138.87 ± 15.77	14.61	<0.01
PPBS (mg/dL)	276.32 ± 36.10	277.22 ± 35.93	2.31	<0.05	276.62 ± 35.85	251.40 ± 31.32	12.51	<0.01
HBA1C (%)	9.14 ± 0.33	9.12± 0.34	2.08	<0.05	9.11 ± 0.36	8.86 ± 0.33	10.19	<0.01
Cholesterol (mg/dL)	230.27 ± 23.05	228.95 ± 23.74	1.09	>0.05	230.28 ± 23.06	210.55 ± 20.57	23.62	<0.01
Triglyceride (mg/dL)	166.85 ± 20.76	165.93 ± 20.89	3.44	<0.01	166.87 ± 20.77	144.70 ± 19.88	28.06	<0.01
LDL (mg/dL)	166.85 ± 22.85	115.93 ± 22.77	2.42	<0.05	116.88 ± 22.82	103.77 ± 20.87	19.80	<0.01
HDL (mg/dL)	36.47 ± 5.23	35.93 ± 5.66	2.65	<0.05	36.40 ± 5.29	37.50 ± 4.99	-6.63	<0.01
SBP (mm Hg)	133.27 ± 9.22	132.18 ± 9.39	1.97	<0.05	133.13 ± 9.06	113.63 ± 9.01	38.65	<0.01
DBP (mm Hg)	86.57 ± 7.13	85.73 ± 6.01	3.05	<0.01	86.47 ± 7.10	82.63 ± 3.38	4.79	<0.01

Both vestibular exercises and yoga demonstrated significant reductions in PPBS levels. In both cases, the p-value was less than 0.01. This suggests that both interventions are effective in improving post-meal glucose control (Tables 1, 3).

Both the vestibular exercises and yoga groups exhibited slight reductions in HbA1c, which reflects long-term glucose control. While the reductions were modest, they were statistically significant.

Notably, the yoga group showed a greater decrease in HbA1c (from an average of 9.11 to 8.86) compared to the control group, with a highly significant p-value of less than 0.01. This indicates that yoga may have a more substantial impact on long-term glucose regulation (Tables 1, 3).

The vestibular exercise group experienced a substantial reduction in total cholesterol and low-density lipoprotein (LDL) cholesterol, both of which had highly significant p-values of less than 0.01.

Yoga, on the other hand, not only significantly reduced total cholesterol and LDL but also lowered triglycerides and increased high-density lipoprotein (HDL) cholesterol, with highly significant p-values (less than 0.01) for all these changes. These results suggest that yoga has a broader positive effect on lipid profiles (Tables 1, 3).

Both systolic and diastolic blood pressures were significantly reduced in both the vestibular exercise and yoga groups (p < 0.01). This indicates that both interventions contribute to improved blood pressure control.

Notably, the yoga group exhibited a particularly prominent decline in systolic blood pressure (from 133.13 to 113.63) with a highly significant p-value of less than 0.01. This suggests that yoga may have a strong influence on systolic blood pressure reduction (Tables 1, 3).

The yoga group demonstrated a significant reduction in BMI (from an average of 27.35 to 24.95), with a highly significant p-value of less than 0.01 (Table 4).

**Table 4:** Baseline anthropometric parameters after three months among the control group and yoga group. BMI: body mass index, SD: standard deviation. The results are considered statistically significant if the p-value is less than 0.05 and highly statistically significant if the p-value is less than 0.01.

Parameters	Control (before) (n=60) mean±SD	Control (after) (n=60) mean±SD	T value	P value	Yoga (before) (n=60) mean±SD	Yoga (after) (n=60) mean±SD	T value	P value
BMI (kg/m²)	27.40 ± 2.73	27.32 ± 2.54	1.21	>0.05	27.35 ± 2.61	24.95 ± 2.44	24.33	<0.01
Body fat %	28.11 ± 3.49	27.95 ± 3.47	0.479	>0.05	27.95 ± 3.47	25.61 ± 3.48	34.67	<0.01
Waist circumference (cm)	100.14 ± 6.47	99.99 ± 6.51	1.48	>0.05	100 ± 6.31	96.05 ± 6.54	14.29	<0.01
Hip circumference (cm)	103.63 ± 5.20	103.52 ± 5.15	1.49	>0.05	103.58 ± 5.14	100.86 ± 4.99	12.10	<0.01
Waist–hip ratio	0.966 ± 0.035	0.965 ± 0.035	0.37	>0.05	0.967 ± 0.0359	0.965 ± 0.035	1.53	<0.05
Arm circumference (cm)	13.94 ± 3.40	13.79 ± 3.45	1.19	>0.05	13.88± 3.41	11.91 ± 3.22	23.04	<0.01
Thigh circumference (cm)	22.22 ± 4.12	22.02 ± 4.16	2.11	<0.05	22.13 ± 4.18	20.10 ± 4.24	24.13	<0.01

The combined vestibular exercise and yoga group exhibited the most substantial reduction in BMI (from an average of 27.34 to 23.62) with a highly significant p-value of less than 0.01. This indicates that the combination of these interventions had the most pronounced effect on BMI reduction (Table 5).

**Table 5:** Baseline anthropometric parameters after three months among the control group and vestibular exercise along with yoga (VE + yoga) group. BMI: body mass index, SD: standard deviation. The results are considered statistically significant if the p-value is less than 0.05 and highly statistically significant if the p-value is less than 0.01.

Parameters	Control (before) (n=60) mean ± SD	Control (after) (n=60) mean ± SD	T value	P value	Ve+ yoga (before) (n=60) mean ± SD	Ve + yoga (after) (n=60) mean ± SD	T value	P value
BMI (kg/m²)	27.40 ± 2.73	27.32 ± 2.54	1.21	>0.05	27.34 ± 2.54	23.62 ± 1.64	15.88	<0.01
Body fat %	28.11 ± 3.49	27.95 ± 3.47	0.479	>0.05	28.24 ± 3.57	21.77 ± 3.48	11.50	<0.01
Waist circumference (cm)	100.14 ± 6.47	99.99 ± 6.51	1.48	>0.05	100.18 ± 6.47	92.43 ± 4.32	11.50	<0.01
Hip circumference (cm)	103.63 ± 5.20	103.52 ± 5.15	1.49	>0.05	103.56 ± 5.17	96.75 ± 4.49	17.40	<0.01
Waist–hip ratio	0.966 ± 0.035	0.965 ± 0.035	0.37	>0.05	0.967 ± 0.0347	0.956 ± 0.0454	1.81	0.074
Arm circumference (cm)	13.94 ± 3.40	13.79 ± 3.45	1.19	>0.05	13.87 ± 3.41	11.42 ± 3.05	14.60	<0.01
Thigh circumference (cm)	22.22 ± 4.12	22.02 ± 4.16	2.11	<0.05	22.15 ± 4.17	19.36 ± 4.16	11.15	<0.01

Both waist and hip circumferences decreased significantly in the yoga group and the combined group (p < 0.01) (Tables 2, 4).

Notably, the combined intervention (vestibular exercise and yoga) resulted in the most significant reductions in waist and hip circumferences (Table 5). This suggests that the combination of these interventions had a greater impact on body composition.

Across all groups, there were minimal changes in the waist-hip ratio. However, the combined intervention group showed a borderline significant decrease (p = 0.074). This indicates that the combined intervention may have a slight effect on altering the waist-hip ratio, although it did not reach full statistical significance (Table 5).

Both arm and thigh circumferences showcased reductions with yoga and the combined intervention, and these reductions were statistically significant (p < 0.01). This suggests that both yoga and the combined approach have a positive influence on body measurements in these areas (Table 5).

## Discussion

In this study, we explored the combined impact of vestibular stimulation activities and yoga on the management of T2DM by assessing their effects on metabolic and anthropometric parameters. The findings of our investigation can be contextualized in relation to previous studies conducted by various authors and their implications for diabetes management.

The observed significant reductions in FBS levels following both vestibular exercises and yoga resonate with the findings of Chimkode et al., emphasizing the beneficial effects of yoga on blood sugar levels [16]. These outcomes corroborate the notion that both interventions independently contribute to glycemic control.

Our study mirrored the favorable impacts of exercise and yoga on post-meal glucose control, aligning with the research of Mann et al., which discusses improved insulin sensitivity in response to different exercise modalities [17]. While both vestibular stimulation activities and yoga showed modest reductions in HbA1c levels, yoga outperformed the control group, supporting previous studies done by Raveendran et al., which underscore yoga's efficacy in enhancing long-term glucose regulation [18].

Our findings regarding lipid profiles resonate with Goldberg and Wilson's research [19], which recognizes exercise prescription as a critical component in managing type 2 diabetes and its associated lipid abnormalities. Furthermore, yoga's comprehensive impact on lipid profiles aligns with Manchnada et al.'s research [20].

Both interventions resulted in significant reductions in systolic and diastolic blood pressures, consistent with the known effects of physical activity and yoga on blood pressure regulation by Thangasami et al. [21]. The substantial decline in systolic blood pressure following yoga intervention echoes the findings of Han et al. [22].

The reduction in BMI observed in the yoga group and the combined intervention group echoes the findings of Prasad et al. [23]. Reductions in body fat percentage were noted in both the yoga group and the combined intervention group, affirming the potential of these interventions to reduce body fat by Liu et al. [24].

Our study aligns with earlier research emphasizing the benefits of exercise and yoga on body composition and waist-hip measurements by Kumar Goothy et al. [25]. The substantial changes noted in waist and hip circumferences following the combined intervention echo these findings.

Although minimal changes were observed across all groups, the borderline significant decrease in the combined intervention group suggests a potential effect on altering the waist-hip ratio, as noted by Sailesh et al. [26]. Both arm and thigh circumferences exhibited reductions in the yoga and combined intervention groups, reinforcing the potential benefits of these interventions on body measurements by Kumar Goothy et al. [25].

In comparing our findings with those of previous authors, it becomes evident that our study contributes to the growing body of evidence supporting the positive effects of yoga and vestibular stimulation activities on various aspects of metabolic and anthropometric parameters. Furthermore, our research extends this understanding by exploring the combined impact of these interventions, hinting at their potential synergistic effects in diabetes management.

However, it is vital to acknowledge the limitations of this study, including the reliance on self-reported data and the relatively short-term nature of the interventions. External factors such as diet and lifestyle changes, which were not controlled for, may have influenced the outcomes.

## Conclusions

Our investigation underscores the significance of combining vestibular stimulation activities and yoga in the management of T2DM. The results emphasize the potential benefits of these interventions in improving metabolic and anthropometric parameters, offering a promising avenue for further research and clinical applications in diabetes care.
